# The role of S-1 in the treatment of gastric cancer

**DOI:** 10.1038/sj.bjc.6604332

**Published:** 2008-03-25

**Authors:** T Kubota

**Affiliations:** 1 Center for Comprehensive and Advanced Medicine, Keio University Hospital, 35 Shinanomachi, Shinjuku-ku, Tokyo 160-8582, Japan

**Keywords:** gastric cancer, adjuvant therapy, S-1

## Abstract

S-1 is a potent antitumour drug in gastric cancer. After years of disagreement about the utility of chemotherapy for advanced gastric cancer, several studies have recently demonstrated the efficacy of S-1 in both the adjuvant and primary settings. In this Minireview, the value of S-1 in the treatment of gastric cancer is discussed.

S-1 is a novel oral anticancer drug composed of tegafur (FT), 5-chloro-2, 4-dihydroxypyridine (gimestat [CDHP]), and oteracil potassium (Oxo) in a molar ratio of 1 : 0.4 : 1. This agent was designed to enhance the oral efficacy of FT, a prodrug of 5-fluorouracil (5-FU). CDHP inhibits the activity of dihydropyrimidine dehydrogenase (DPD), an enzyme that degrades 5-FU, with about 180-fold more potency than uracil, thereby maintaining prolonged blood and tumour 5-FU concentrations. Oxo is distributed in the gastrointestinal tract at a high concentration following oral administration, and it prevents phosphorylation (i.e., activation) of 5-FU by inhibiting the effect of orotate phosphoribosyl transferase (OPRT). S-1 therefore has improved tumour-selective toxicity compared to 5-FU alone due to the actions of CDHP and Oxo as shown in Figure 1 (Shirasaka *et al*, 1996).

Pharmacokinetic analyses have supported this potent antitumour activity of S-1. In a rodent model, S-1 doses that were equitoxic to UFT doses resulted in higher 5-FU levels, as revealed by the following pharmacokinetic measurements: single-day area under the curve (AUC_0–24_) of 5-FU in plasma (3.5-fold), AUC_0–24_ of 5-FU incorporated into tumour RNA (1.3-fold), and tumour thymidylate synthase inhibition rate (∼20%). In a clinical pharmacology study in 12 patients that examined blood concentrations of 5-FU following twice-daily administration of S-1 40 mg m^−2^ after meals, 5-FU blood concentrations ranged from 60–200 ng ml^−1^ (Hirata *et al*, 1999). These results suggested that oral S-1 administration should result in a similar concentration of 5-FU as intravenous 5-FU administration, and should therefore have equivalent antitumour activity.

Herein, the role of S-1 in the treatment of gastric cancer is discussed, with particular emphasis on its use as postsurgical adjuvant therapy.

## PHASE II S-1 STUDIES

Two phase II studies were conducted to confirm the antitumour activity of S-1 in gastric cancer patients. In the first study, 51 patients with advanced gastric cancer without previous chemotherapy were enroled, 50 of whom were included in the efficacy and safety analyses (Sakata *et al*, 1998). S-1 was administered orally two times daily for 28 days, followed by a 2-week rest. Doses were assigned according to patient body surface area: <1.25 m^2^, 40 mg; 1.25–1.5 m^2^, 50 mg; and ⩾1.5 m^2^, 60 mg. The overall response rate (ORR) was 44% (19 out of 43), with a 95% confidence interval (CI) of 30–59%. The median survival time (MST) in all patients was 207 days, with 1- and 2-year survival rates of 36.0 and 14.0%, respectively. The second phase II study (Koizumi *et al*, 2000) enroled 51 patients with advanced gastric cancer. S-1 80 mg m^−2^ per day was administered orally two times daily. A single course consisted of consecutive administration for 28 days followed by a 2-week rest; administration was repeated over four courses. The ORR was 49% (25 out of 51), with a 95% CI of 35.9–62.3%.

Adverse events were generally mild in both phase II studies. In the first trial, grade 3 adverse reactions included anaemia in two patients and leukopenia, neutropenia, and diarrhoea in one patient each. No grade 4 or unexpected adverse events were observed (Sakata *et al*, 1998). In the second trial, the overall incidence of adverse events was 78% (40 out of 51 patients), while the incidence of grade 3 and 4 events was 20% and included anaemia, leukopenia, granulocytopenia, diarrhoea, malaise, and proteinuria (Koizumi *et al*, 2000). No serious unexpected adverse events were observed in either study.

## PHASE III S-1 STUDIES

Based on the encouraging results of a phase I/II study that evaluated S-1+DDP, Koizumi *et al* (2008) conducted a randomised, controlled, open-label, parallel, multicenter phase III study (SPIRITS trial) comparing S-1 alone with S-1+DDP. Patients with advanced/recurrent gastric cancer who had not previously received S-1 were randomised to receive either S-1 40 mg m^−2^ two times daily for 28 days followed by a 2-week rest or S-1 40 mg m^−2^ two times daily for 21 days followed by a 2-week rest+intravenous DDP (60 mg m^−2^) on day 8. Between March 2002 and November 2003, 305 patients were randomised, with 299 patients eligible for safety and efficacy evaluation. The 2-year MST was 335.5 days for S-1 alone and 396.0 days for the combination, and OS was significantly longer in the combination arm than in the S-1 monotherapy arm (*P*=0.0366). The RR was also higher in the combination therapy arm (54.0%) than in the S-1 arm (31.1%). The study authors concluded that this regimen should be regarded as one of the first-line standard treatments for advanced/recurrent gastric cancer.

Imamura *et al* (2008) conducted a multicenter, randomised phase III trial comparing irinotecan (CPT-11)+S-1 (IRIS) to S-1 alone in advanced gastric cancer patients. Patients were randomised to either S-1 40 mg m^−2^ two times daily for 28 days followed by a 14-day rest or S-1 40 mg m^−2^ two times daily for 21 days+CPT-11 80 mg m^−2^ on days 1 and 15 followed by a 2-week rest. An interim analysis revealed an ORR of 26.9% for the S-1 monotherapy arm and 41.5% for the IRIS arm (*P*=0.035), however IRIS didn't show statistically significant superiority to S-1 alone in OS.

The combination of docetaxel and S-1 has been evaluated in two phase II studies. In one study, Yoshida *et al* (2006) assessed the efficacy and toxicity of docetaxel and S-1 for patients with advanced/recurrent gastric cancer. Patients received intravenous docetaxel 40 mg m^−2^ on day 1 and S-1 40 mg m^−2^ two times daily for 14 days followed by a 1-week rest. The ORR was 56.3% (95% CI, 38–66%). The MST was 14.3 months, and median time to tumour progression was 7.3 months. No grade 4 nonhaematologic toxicities were reported, and all treatment-related toxicities were resolved. In the second phase II study (Yamaguchi *et al*, 2006), treatment consisted of intravenous docetaxel 50 mg m^−2^ on day 1 and S-1 40 mg m^−2^ two times daily for 14 days, followed by a 2-week rest. The maximum tolerated dose of docetaxel was determined to be 50 mg m^−2^, and the recommended dose was reduced to 40 mg m^−2^. In the 46 patients who received treatment, the ORR and estimated median OS were 46% (95% CI, 31–61%) and 14.0 months, respectively. The most common grade 3 of 4 toxicity was neutropenia, which was predictable and manageable. These two phase II studies indicated that this regimen results in extended survival despite a modest ORR, and that toxicity is manageable. A phase III study (JACCRO GC-03/START trial) is currently underway to compare S-1 monotherapy with S-1+docetaxel 40 mg m^−2^ in Japanese and Korean patients with inoperable advanced or recurrent gastric cancer.

## THE N-SAS-GC TRIAL: USEFULNESS OF UFT AS ADJUVANT THERAPY AFTER GASTRIC SURGERY

UFT is the immediate predecessor of S-1. When this compound is administered to patients, the degradation of tegafur is competitively inhibited by uracil, a substrate of DPD, in a reversible manner. Nakajima *et al* (2007) evaluated the survival benefit of adjuvant UFT administration following curative resection of advanced gastric cancer in which the tumour depth of invasion is limited within the subserosal layer and the degree of lymph node metastasis corresponds to N1-2 according to the Japanese Classification of Gastric Carcinoma. Eligibility criteria included curative gastrectomy with D2 or greater lymph node dissection, ECOG performance status (PS) 0–2, and no prior chemotherapy. Patients were randomised in a 1 : 1 ratio to surgery plus adjuvant chemotherapy or surgery alone. The UFT arm consisted of oral UFT 360 mg m^−2^ per day for 5 days with a 2-day rest, beginning 6 weeks after surgery and ending 16 months after surgery. The primary end point was OS, and relapse-free survival was a secondary end point. Although enrolment of 244 patients in each arm was initially planned, the study was closed after accrual of 190 patients, due to slow recruitment primarily caused by the wide introduction of S-1 into clinics. The planned second interim analysis was conducted in November 2004 after a median follow-up of 3.8 years (3 years after registration was closed). A total of 188 patients were randomised to the UFT arm (*n*=93) or the surgery-alone arm (*n*=95). Patients in the UFT arm experienced significantly longer 5-year OS (86 *vs* 73%, *P*=0.017, hazard ratio [HR]=0.48) and 5-year relapse-free survival (85 *vs* 68%, *P*=0.005, HR=0.44) compared to patients in the surgery-alone arm. Although the planned sample size was not achieved, the results of this study suggested that adjuvant oral fluoropyrimidine monotherapy can provide a definitive survival benefit in advanced gastric cancer patients, with minimal adverse effects.

## ADJUVANT CHEMOTHERAPY FOLLOWING CURATIVE RESECTION OF GASTRIC CANCER: THE ACTS-GC TRIAL

Prior to the use of S-1 in postgastrectomised patients, Kinoshita *et al* (2004) conducted a feasibility study of S-1 in curatively resected gastric cancer patients. S-1 80–120 mg day^−1^ was administered for 4 weeks followed by a 2-week rest, for a total of eight courses. From November 1999 to October 2000, 41 patients from 11 institutions were enroled, and 35 patients were eligible for safety and efficacy analyses. Among the 28 patients who did not experience recurrence, the planned eight courses of S-1 were administered to 17 patients (60.7%). Four patients refused continuous S-1 administration due to subjective symptoms, such as anorexia, during the first course. Although adverse events including neutropenia, leukopenia, elevated total bilirubin, anorexia, general fatigue, diarrhoea, nausea, and stomatitis were observed in >50% patients, no grade 4 adverse events occurred. Thus, postoperative administration of S-1 for 1 year appears to be feasible as adjuvant chemotherapy for gastric cancer.

On the basis of this study, Sakuramoto *et al* (2007) initiated the ACTS-GC trial, which evaluated single-agent S-1 as adjuvant chemotherapy for Japanese patients with stage II/III (Japanese Classification of Gastric Carcinoma) gastric cancer following curative D2 dissection. Inclusion criteria included R0 resection, pathological stage II/III (excluding T1 cases), age 20–80 years, no prior adjuvant treatment, and adequate organ function. Patients were randomly assigned to receive surgery plus S-1 or surgery alone. S-1 80 mg m^−2^ per day was administered for 4 weeks followed by a 2-week rest beginning within 45 days of surgery and ending 1 year after surgery. The primary end point was OS. Two interim analyses were planned at 1 and 3 years after enrolment was completed. Between October 2001 and December 2004, 1059 patients were accrued, with 529 randomised to the S-1 treatment arm and 530 randomised to the surgery-alone arm; no significant differences in baseline characteristics existed between the two groups. When the first interim analysis was conducted in June 2006 using the survival data acquired through December 2005, the HR of death for S-1 to surgery alone was 0.57 (95% CI, 0.40–0.81; *P*=0.0016). On the basis of these results, the data and safety monitoring committee recommended halting this trial. When the final analysis was conducted in September 2006, using data through June 2006, 3-year OS was 80.5% (95% CI, 76.6–84.4%) for S-1 patients and 70.1% (95% CI, 65.5–74.6%) for patients who received surgery alone. The HR of death for S-1 was 0.68 (95% CI, 0.52–0.87; *P*=0.0024). A total of 65.8% patients completed S-1 therapy. The results of this trial demonstrated that adjuvant chemotherapy with S-1 for stage II/III gastric cancer is feasible and effective, and suggested that adjuvant S-1 should be adopted as the standard treatment following curative D2 gastric dissection.

## DISCUSSION

Results from several phase II studies in advanced gastric cancer were reported in the Japanese Guidelines for Treatment of Gastric Cancer (2nd Edition) (Japanese Gastric Cancer Association, 2004), with median response rates ranging from 41 to 78% and MSTs ranging from 7 to 12.6 months, which were markedly higher than the MST of 3.6 months observed for best supportive care. The JCOG9912 trial compared the efficacy and toxicity of three regimens: 5-FU, CPT-11+CDDP (CP), and S-1 (Boku *et al*, 2007). In this study, the MSTs were 9.0, 12.1, and 10.5 months for 5-FU, CP, and S-1, respectively, with S-1 demonstrating significant non-inferiority to 5-FU, while CP did not show statistically significant superiority to 5-FU. On the basis of these results, S-1 monotherapy was adopted as the standard cancer chemotherapy regimen for inoperable and recurrent gastric cancer in Japan. However, after the SPIRITS trial (Koizumi *et al*, 2008) revealed the superiority of S-1+CDDP compared with S-1 alone, the preferred chemotherapy regimen for chemo-naive advanced gastric cancer patients in Japan is now considered to be S-1+CDDP.

After many years of controversy regarding the utility of postoperative gastric cancer chemotherapy, the ACTS-GC study has demonstrated the efficacy of S-1 monotherapy as adjuvant treatment following D2 dissection of stage II/III gastric cancer. However, there appears to be ethnic differences in the pharmacokinetic profile of S-1. Chu *et al* (2004) have evaluated S-1 given once daily for 28 days every 5 weeks for the determination of maximum tolerated dose, pharmacokinetics, and anticancer activity for non-Japanese patients. The maximum tolerated dose was defined as the highest dose at which fewer than two of the first six new patients experience dose-limiting toxicity. When 20 patients were treated with S-1 at three dose levels ranging from 50–70 mg m^−2^ per day, diarrhoea associated with abdominal discomfort and cramping was the principal dose-limiting toxicity. Nausea, vomiting, mucositis, fatigue, and cutaneous effects were also observed, but were not dose-limiting; bone marrow suppression was also not dose-limiting. These results contradict those observed in Japanese patients, who tend to develop dose-limiting bone marrow toxicity prior to experiencing gastrointestinal adverse events. This might be due to the different activity of OPRT, which activates 5-FU in the bowel mucosa, in Japanese patients. Patients with higher OPRT activity might be expected to experience a higher incidence of gastrointestinal adverse effects prior to bone marrow toxicity, even after similar Oxo dosing. On the basis of this possible pharmacokinetic difference observed between Japanese and non-Japanese patients, Chu *et al* (2004) recommended S-1 50 mg m^−2^ per day administered once daily for 28 consecutive days every 5 weeks, which is significantly lower than 80 mg m^−2^ per day for 4 weeks, the dose used in the ACTS-GC study. This lower S-1 dose, which is used outside of Japan, was used in the FLAGS study, a phase III advanced gastric cancer study in which S-1 (50 mg m^−2^ per day, day 1–21)+DDP (75 mg m^−2^ per day, day 1) was compared to 5-FU (1000 mg m^−2^ per day, day 1–5)+DDP (100 mg m^−2^ per day, day 1), which is considered to be the standard therapy for advanced gastric cancer. The results of the FLAGS study will be reported in 2008.

S-1 is now considered to be a key drug for inoperable and recurrent gastric cancer, as well as adjuvant cancer chemotherapy after D2 gastrectomy in Japan. While combinations of S-1 with DDP, CPT-11, and docetaxel will be confirmed by phase III studies, how to best sequence these regimens following late recurrences after S-1 adjuvant therapy remains unknown. While the commonly used standard 5-FU+DDP regimen will be compared to S-1+DDP in the FLAGS trial, these doublets should also be compared to triplet regimens such as docetaxel, DDP, and 5-FU (DCF) (Van Cutsem *et al*, 2006). In addition, recently introduced molecularly targeted therapies, including monoclonal antibodies and tyrosine kinase inhibitors, are excellent candidates for combining with conventional antitumour agents, as has been successfully done in colorectal cancer.

## Figures and Tables

**Figure 1:**
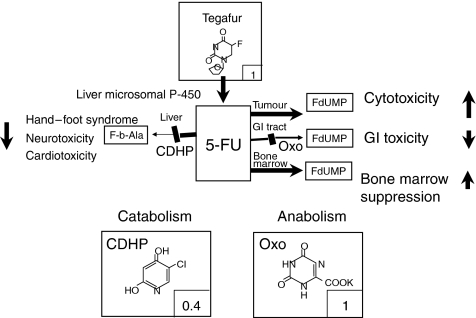
The mode of action of S-1. S-1 is composed of tegafur (FT), 5-chloro-2, 4-dihydroxypyridine (gimestat [CDHP]), and oteracil potassium (Oxo) in a molar ratio of 1 : 0.4 : 1 to enhance the oral efficacy of FT, a prodrug of 5-fluorouracil (5-FU). CDHP inhibits the activity of dihydropyrimidine dehydrogenase (DPD), an enzyme that degrades 5-FU, with about 180-fold more potency than uracil. Oxo is distributed in the gastrointestinal tract at a high concentration following oral administration, and it prevents phosphorylation (i.e., activation) of 5-FU by inhibiting the effect of orotate phosphoribosyl transferase (OPRT).
